# *PIK3R2*-related megalencephaly syndrome: expanded phenotypic spectrum associated with craniosynostosis

**DOI:** 10.1186/s13023-026-04666-y

**Published:** 2026-10-22

**Authors:** Evangelos Digkas, Izabella Baranowska Körberg, Evangelos Mourtos, Daniel Nowinski, Christos Aravidis

**Affiliations:** 1 Department of Immunology, Genetics and Pathology, Uppsala University, Uppsala, Sweden; 2 Radiology, Department of Pediatric Radiology, Karolinska University Hospital, Stockholm, Sweden; 3 Department of Surgical Sciences, Plastic surgery, Uppsala University, Uppsala, Sweden; 4 Department of Laboratory Medicine, Clinical Genetics Department, Faculty of Medicine and Health, Örebro University Hospital, Örebro University, Örebro, Sweden

**Keywords:** PIK3R2, MPPH syndrome, Craniosynostosis, Megalencephaly, Polymicrogyria, Whole exome sequencing

## Abstract

**Background:**

Megalencephaly-polymicrogyria-polydactyly-hydrocephalus (MPPH) syndrome is a rare neurodevelopmental disorder caused by activating variants in the PI3K-AKT-mTOR signaling pathway, most frequently in PIK3R2. The recurrent variant c.1117G > A (p.Gly373Arg) has been associated with classical MPPH features including megalencephaly, bilateral polymicrogyria, hydrocephalus, and polydactyly. However, the phenotypic spectrum is still expanding.

**Methods:**

We describe a 7-year-old girl with developmental delay, epilepsy, and structural brain anomalies. Trio-based whole exome sequencing followed by Sanger validation was performed. Clinical and radiological evaluations were conducted, including brain MRI, CT, and ophthalmological assessment.

**Results:**

The patient exhibited congenital megalencephaly, bilateral perisylvian and parietotemporal polymicrogyria, and hypotonia. She lacked polydactyly but presented with complex bicoronal and sagittal craniosynostosis, representing a potential novel association with this variant. Imaging revealed signs of chronic intracranial hypertension, including optic nerve tortuosity, papilledema, pituitary flattening, and gyral impressions. A heterozygous de novo PIK3R2 c.1117G > A (p.Gly373Arg) variant was identified and classified as pathogenic.

**Conclusion:**

This report broadens the phenotypic spectrum of PIK3R2-related MPPH syndrome by describing a potential novel association with craniosynostosis, accompanied by radiologic signs of intracranial hypertension. The findings support the inclusion of PIK3R2 in genetic work-ups for patients with cortical malformations, megalencephaly, and syndromic presentations—even in the absence of polydactyly.

## Introduction

Megalencephaly-polymicrogyria-polydactyly-hydrocephalus (MPPH) syndrome is a rare neurodevelopmental disorder first described in 2004 and characterized by a constellation of findings including congenital megalencephaly, bilateral perisylvian polymicrogyria (BPP), hydrocephalus or ventriculomegaly, postaxial polydactyly, hypotonia, intellectual disability, and early-onset epilepsy [1, 2]. MPPH is caused by activating germline or somatic variants in core components of the PI3K-AKT-mTOR signaling pathway, particularly PIK3R2, AKT3, and CCND2 [3, 4]. These variants lead to dysregulated cellular growth and cortical development due to constitutive signaling through this pathway. PIK3R2, the most frequently mutated gene in MPPH, encodes the p85β regulatory subunit of class IA phosphoinositide 3-kinases (PI3Ks), which modulate AKT activation through phosphorylation and control downstream targets such as mTOR complexes [5–7]. A murine model harboring the PIK3R2 p.G367R variant—homologous to the human p.Gly373Arg—recapitulates key features of MPPH, including megalencephaly and increased cortical thickness, further supporting its pathogenicity [8].

Variants in PIK3R2 are typically missense and lead to gain-of-function effects. To date only 27 variants have been reported [9, 10] (accessed May 2025). The most commonly reported pathogenic variant, c.1117G > A (p.Gly373Arg), has been shown to increase PI3K-AKT signaling and cortical overgrowth in animal models [8]. Although the phenotypic spectrum of MPPH overlaps with that of megalencephaly-capillary malformation (MCAP) syndrome, which is caused by PIK3CA mutations, the presence of vascular malformations distinguishes MCAP from MPPH [2, 11].

Since its original description, fewer than 70 cases of MPPH have been reported worldwide, with only a subset harboring the PIK3R2 gene [12]. These cases demonstrate variable expressivity, with some lacking hallmark features such as polydactyly or hydrocephalus. Craniosynostosis has not previously been reported in association with MPPH, raising the question of whether it is a coincidental finding or a potential previously underrecognized part of the phenotype.

Here, we present a detailed clinical, neuroimaging, and molecular characterization of a 7-year-old girl with megalencephaly, polymicrogyria, developmental delay, and epilepsy, who was found to carry the recurrent pathogenic PIK3R2 c.1117G > A (p.Gly373Arg) variant. Notably, she also exhibited complex craniosynostosis affecting the coronal and sagittal sutures, representing a potential novel phenotypic association with MPPH. This case contributes to a broader understanding of PIK3R2-related disease and underscores the importance of early genetic evaluation in children with structural brain anomalies and neurodevelopmental delay.

## Materials and methods

### Patient

The proband is a 7-year-old girl of Iraqi origin who was referred to the Department of Clinical Genetics at Uppsala University Hospital for evaluation of developmental delay and suspected craniosynostosis. Clinical, radiological, and genetic data were collected as part of the diagnostic work-up. MRI and CT imaging confirmed polymicrogyria and complex craniosynostosis. There was no evidence of polydactyly or vascular malformations.

### DNA extraction and whole exome sequencing

Peripheral blood samples were collected from the patient and her parents. Genomic DNA was extracted using an automated system (QuickGene, QIAGEN Inc.) according to standard protocols. DNA quality and concentration were verified prior to sequencing.

Clinical whole exome sequencing (WES) was performed using a trio-based approach. In brief, 250 ng of genomic DNA from each individual (patient, mother, and father) was used for library preparation with the Twist Human Core Exome kit (Twist Bioscience), targeting coding exons and flanking splice junctions. Enriched libraries were sequenced on an Illumina NextSeq500 platform with 150 bp paired-end reads.

Bioinformatic processing and variant calling were conducted using the Bcbio Nextgen v0.8.9 pipeline. Sequence reads were aligned to the human reference genome (GRCh37/hg19) using BWA v0.7.12, and variant calling was performed with GATK HaplotypeCaller (v3.2.4). Quality control metrics included FastQC 0.11.3, Picard HsMetrics 1.96, and GATK Depth of Coverage.

Variant annotation and prioritization were performed using Alissa Interpret (Agilent Technologies Inc.). Variants were classified according to the American College of Medical Genetics and Genomics (ACMG) and Association for Molecular Pathology (AMP) guidelines. The pathogenic variant identified was subsequently confirmed by Sanger sequencing in all trio samples. Sequencing reactions were run in both forward and reverse directions using BigDye Terminator v3.1 on a 3500 XL Genetic Analyzer (Applied Biosystems) and analyzed using SequencePilot v4.3.1 (JSI Medical Systems GmbH).

The average coverage of the exome was > 95% at ≥ 10× read depth, and > 98% of the targeted coding regions were successfully captured and analyzed. Non-coding variants and large deletions/duplications were not assessed.

## Results

### Clinical findings

The proband is a 7-year-old girl of Iraqi origin who was referred for genetic evaluation due to suspected craniosynostosis and neurodevelopmental delay. She was born by cesarean section in Iraq due to prenatal suspicion of hydrocephalus and underwent neurosurgical intervention around 6 months of age. She immigrated to Sweden at the age of six years.

At the time of evaluation, the patient presented with congenital megalencephaly and global developmental delay. She exhibited delayed expressive language and was able to speak only a few words in her native language (Arabic) at age seven and could follow simple instructions. Significant expressive language delay was noted. There were no reported difficulties with appetite, sleep, micturition, or defecation. The patient had a history of epilepsy with onset at one year of age, which was well controlled with carbamazepine. Neurological examination revealed hypotonia and mild motor impairment.

**Fig. 1:**
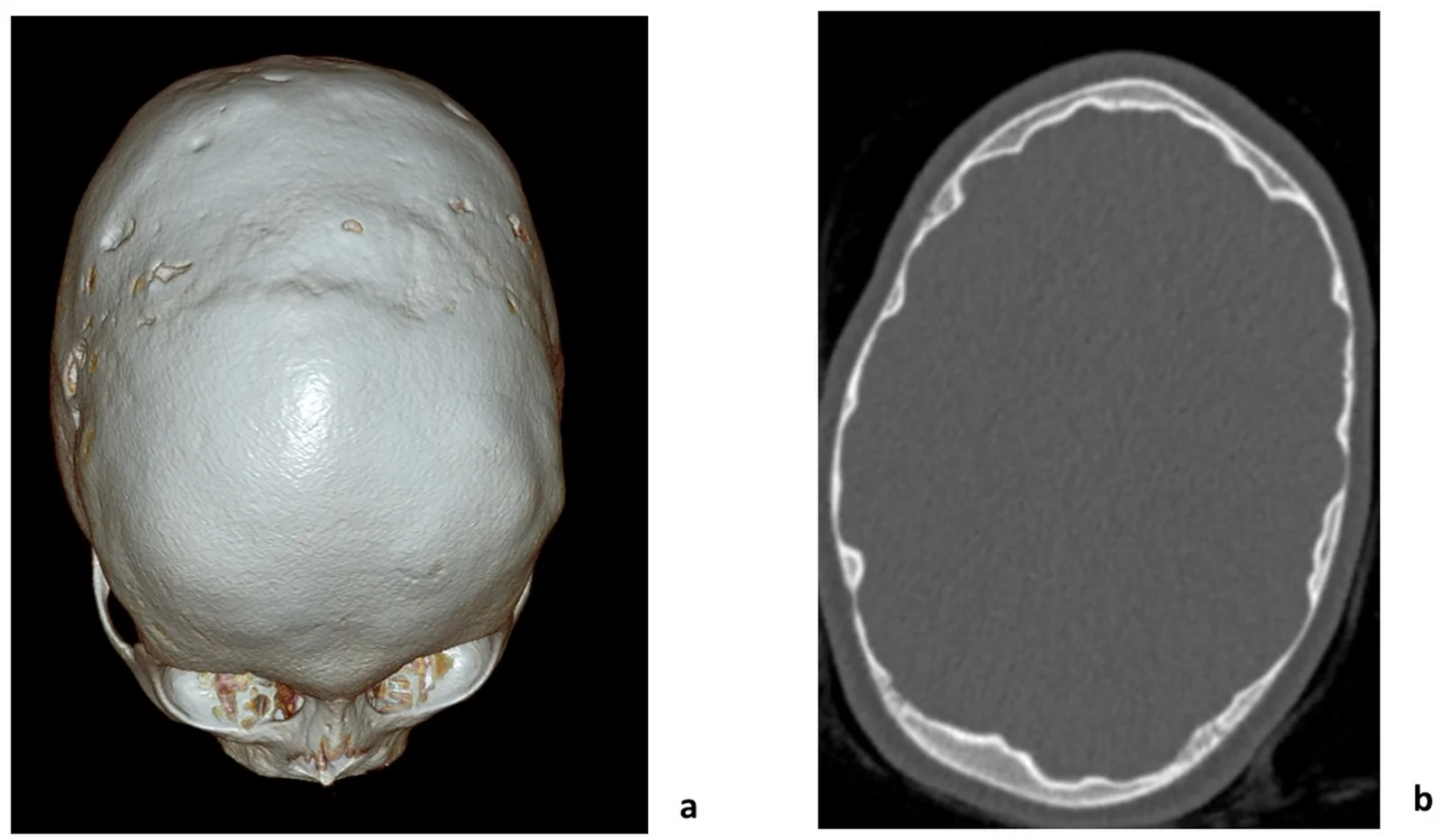
CT images of the skull demonstrating craniosynostosis and signs of elevated intracranial pressure. **(a)** Three-dimensional volume-rendered CT (VRT) image showing combined sagittal and bicoronal craniosynostosis. **(b)** Axial CT image (bone window) showing gyral impressions on the inner table of the skull, suggestive of elevated intracranial pressure

**Fig. 2:**
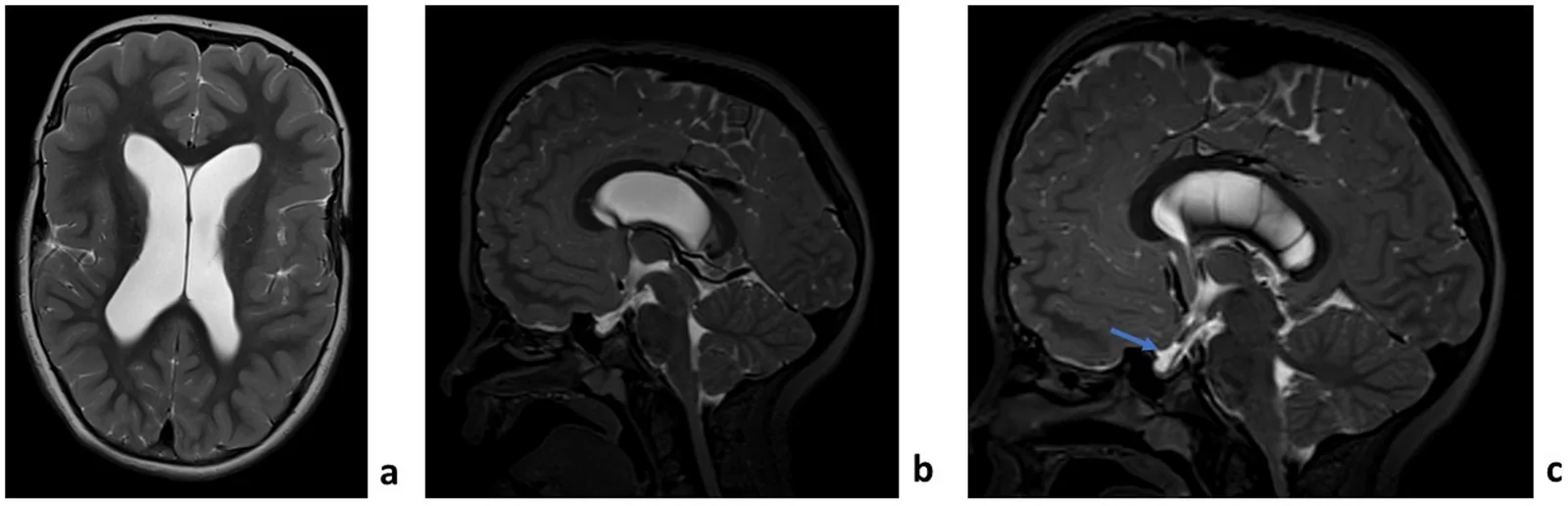
Brain MRI demonstrating ventricular morphology and signs of elevated intracranial pressure. **(a)** Axial T2-weighted MRI image showing moderate enlargement of the lateral ventricles. **(b)** Sagittal T2-weighted MRI showing normal size of the third and fourth ventricles. **(c)** Sagittal T2-weighted MRI image with flattening of the pituitary gland (arrow), consistent with raised intracranial pressure

Cranial CT demonstrated a combined bicoronal and sagittal craniosynostosis (Fig. 1a) and signs of elevated intracranial pressure, including gyral impressions on the inner table of the skull (Fig. 1b). Brain MRI showed moderate enlargement of the lateral ventricles (Fig. 2a), with a small third ventricle and a normally sized fourth ventricle (Fig. 2b), and no periventricular edema—findings consistent with shunt-treated hydrocephalus and no active hydrocephalus. Further MRI findings included extra cerebrospinal fluid surrounding the optic nerves, herniation of the parahippocampal gyrus and uncus, and tapered (narrowed) basal cisterns—indicative of chronically elevated intracranial pressure.

**Fig. 3:**
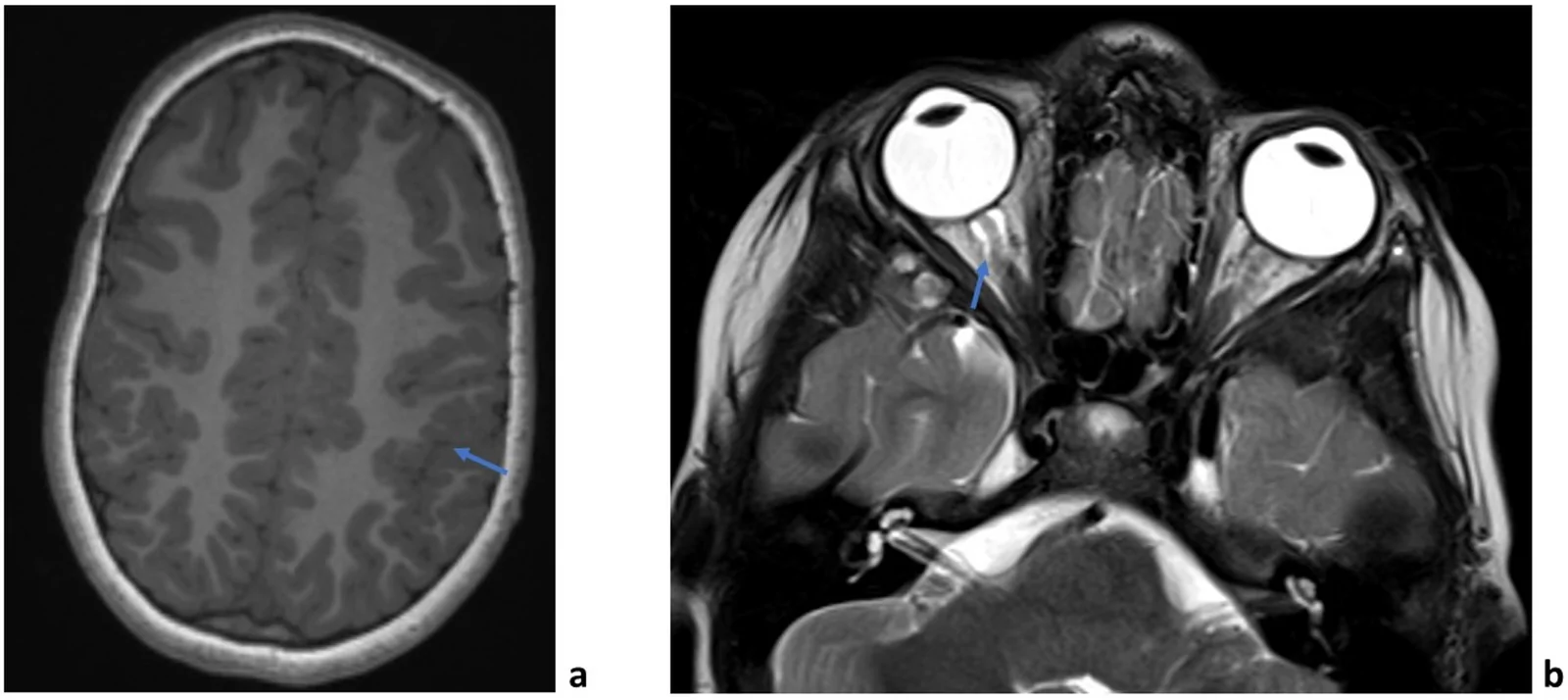
MRI findings of cortical malformation and optic nerve morphology. **(a)** Axial T1-weighted MRI demonstrating polymicrogyria in the left temporoparietal region (arrow). **(b)** Axial T2-weighted MRI of the orbits showing tortuous optic nerves and distension of the perioptic subarachnoid space (arrow)

Additional signs of increased intracranial pressure included flattening of the pituitary gland (Fig. 2c). Axial T1-weighted MRI revealed bilateral polymicrogyria, most prominent in the perisylvian and parietotemporal regions, particularly on the left side (Fig. 3a). The white matter volume appeared globally reduced. Axial T2-weighted MRI showed tortuous optic nerves with distension of the perioptic subarachnoid space (Fig. 3b).

**Fig. 4:**
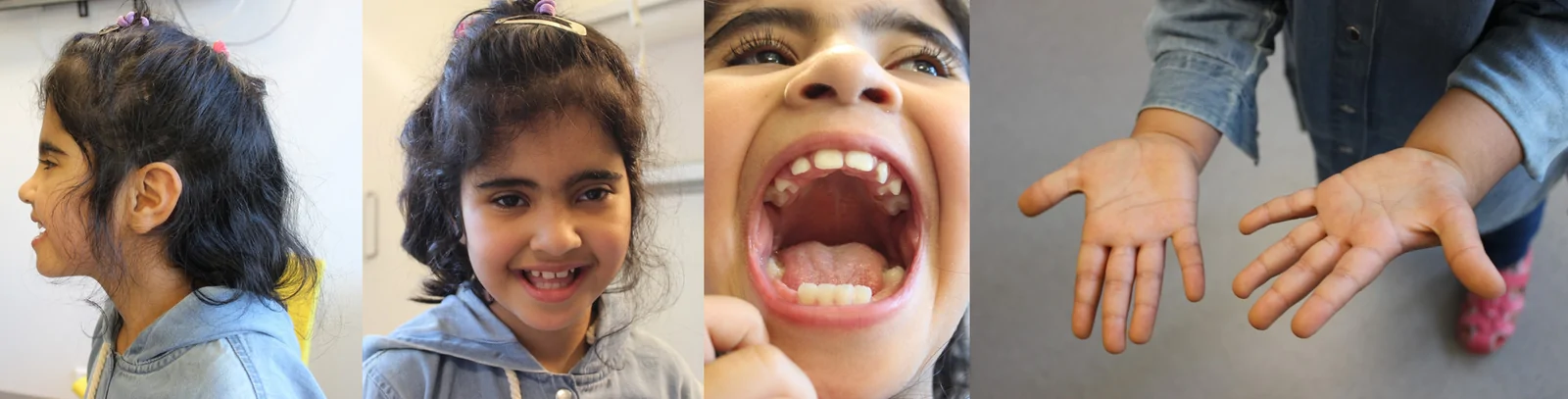
Clinical photograph illustrating craniofacial features including turricephaly, synophrys, down-slanting palpebral fissures, flat philtrum, prominent chin and high-arched palate

Dysmorphic features included turricephaly, synophrys, down-slanting palpebral fissures, a flat philtrum, high-arched palate, a prominent chin, and both low anterior and posterior hairline. Head circumference was enlarged (exact SD not recorded). There were no skeletal anomalies in the extremities and no evidence of polydactyly. Additional features included two café-au-lait macules located on the trunk and increased intermammillary distance. Cardiac and abdominal ultrasound examinations were normal. A clinical photograph illustrating the craniofacial phenotype is shown in Fig. 4. No signs of polydactyly, capillary malformations, or other visible abnormalities were noted on inspection of the extremities. Ophthalmological examination revealed papilledema consistent with elevated intracranial pressure.

### Genetic analysis

Trio-based whole exome sequencing (WES) identified a heterozygous missense variant in the PIK3R2 gene: c.1117G > A (p.Gly373Arg), located in exon 7 (NM_005027.5). The variant affects a highly conserved glycine residue in the regulatory subunit p85β of the PI3K complex and has previously been reported as pathogenic in individuals with megalencephaly-polymicrogyria-polydactyly-hydrocephalus (MPPH) syndrome.

The variant was classified as pathogenic according to ACMG/AMP guidelines based on the following criteria: PS1, PM1, PM2, PP2, and PP3. It is absent from population databases including gnomAD and predicted to be deleterious by multiple in silico tools.

**Fig. 5:**
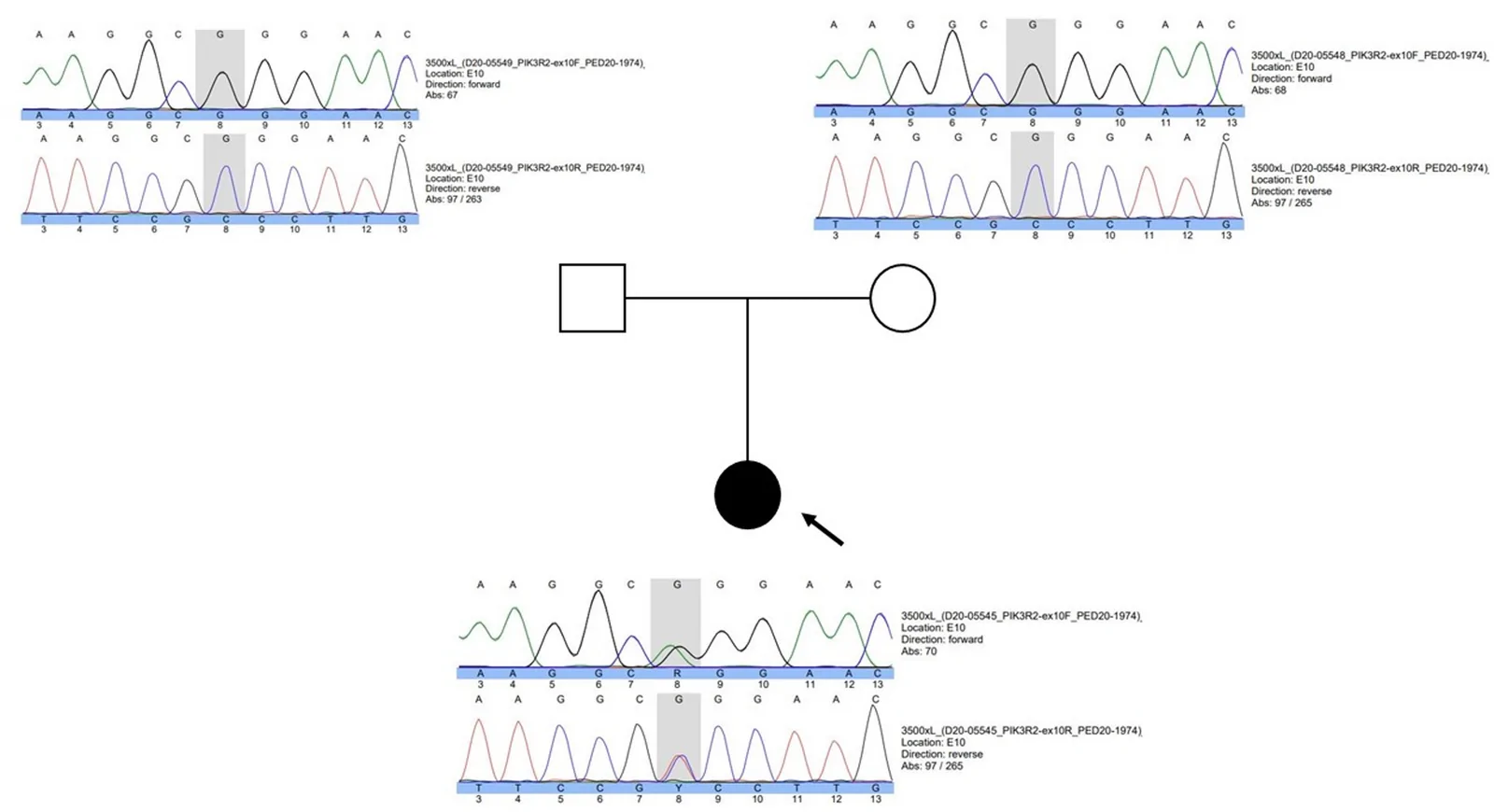
Sanger sequencing chromatograms confirming the heterozygous c.1117G > A (p.Gly373Arg) variant in the PIK3R2 gene in the proband. Forward and reverse reads from the patient are shown in the lower panel, demonstrating a clear heterozygous peak (R) at nucleotide position 1117. The parental sequences (father and mother, upper panels) display only the reference nucleotide (G), consistent with a de novo origin

Sanger sequencing confirmed the presence of the variant in the proband and demonstrated its absence in both parents, supporting a de novo origin (Fig. 5). No other pathogenic or likely pathogenic variants relevant to the phenotype were identified.

## Discussion

The heterozygous missense variant c.1117G > A (p.Gly373Arg) in the PIK3R2 gene is a recurrent, pathogenic variant associated with megalencephaly-polymicrogyria-polydactyly-hydrocephalus (MPPH) syndrome. This variant is among the most commonly reported germline changes in PIK3R2 and has been identified in numerous individuals with developmental brain overgrowth disorders [3, 13]. Functional studies have shown that p.Gly373Arg results in increased PI3K activity and overactivation of the PI3K-AKT-mTOR pathway [3].

The clinical phenotype of individuals harboring this variant typically includes congenital megalencephaly, bilateral perisylvian polymicrogyria, postaxial polydactyly, and variable degrees of hydrocephalus. In a large cohort of individuals with PI3K-AKT-related megalencephaly syndromes, Mirzaa et al. (2015) demonstrated that the p.Gly373Arg variant was strongly associated with the MPPH phenotype. Additional features such as epilepsy, hypotonia, and developmental delay are frequently described as well [14, 15].

The patient described in our study exhibited several hallmark features of MPPH syndrome, including congenital megalencephaly, bilateral perisylvian and parietotemporal polymicrogyria, hypotonia, and global developmental delay. However, she also presented with complex craniosynostosis and signs of chronically elevated intracranial pressure—features that have not been previously reported in association with PIK3R2 mutations. To date, craniosynostosis has been described only in preclinical animal models in association with mTOR pathway dysregulation [16], but has not been reported in patients with MPPH syndrome. Given that PIK3R2 gain-of-function variants activate the PI3K-AKT-mTOR pathway, these experimental findings provide biological plausibility for a potential association between PIK3R2-related pathway activation and craniosynostosis. However, this potential mechanism remains speculative and requires further investigation. To our knowledge, this is the first case of craniosynostosis occurring in a patient with a pathogenic PIK3R2 variant, suggesting a potential novel phenotypic association; however, a causal relationship cannot be established from a single case. The presence of optic nerve tortuosity, papilledema, pituitary flattening and gyral impressions on the skull indicates chronically elevated intracranial pressure. However, in this patient, increased intracranial pressure may be secondary to the complex craniosynostosis and/or the history of hydrocephalus rather than an independent manifestation of PIK3R2-related disease.

Notably, the recurrent p.Gly373Arg variant has also been reported in patients with bilateral perisylvian polymicrogyria without other cardinal features of MPPH syndrome [13], underscoring the variable expressivity of this mutation. Additionally, our patient did not exhibit polydactyly, a common finding in previously described cases [17, 18]. Other clinical manifestations that have been associated with MPPH—such as congenital heart defects, hypothyroidism, renal anomalies, medulloblastoma, and cleft palate [19–21] — were also absent in this case. This observation highlights the phenotypic heterogeneity of germline PIK3R2 mutations and suggests that modifier genes, epigenetic factors, or undetected mosaicism may contribute to the variability in clinical presentation.

Genetic diagnosis in this case was confirmed through trio-based whole exome sequencing followed by Sanger sequencing, which demonstrated a de novo origin of the variant. This provides strong molecular evidence for pathogenicity and strengthens the diagnostic interpretation.

Taken together, this case broadens the clinical spectrum of the PIK3R2 p.Gly373Arg variant and underscores the importance of including this gene in diagnostic panels for children with cortical malformations, megalencephaly, and syndromic neurodevelopmental disorders, even in the absence of more classic findings such as polydactyly. Moreover, the potential association with craniosynostosis warrants further investigation and suggests that attention to skull morphology and clinical signs of increased intracranial pressure may be appropriate during follow-up of patients with PIK3R2-related MPPH syndrome.

## Data Availability

Not applicable. The article describes a potential novel phenotypic association in a previously known diagnosis. All data and materials relevant to this case are provided in the article and are available to the reviewers.
